# Transcriptome and Metabolome Profiling Provide Insights into Flavonoid Synthesis in *Acanthus ilicifolius* Linn

**DOI:** 10.3390/genes14030752

**Published:** 2023-03-20

**Authors:** Zhihua Wu, Zhen Wang, Yaojian Xie, Guo Liu, Xiuhua Shang, Ni Zhan

**Affiliations:** 1 Research Institute of Fast-Growing Trees, Chinese Academy of Forestry, Zhanjiang 524022, China; 2 School of Life Sciences, Langfang Normal University, Langfang 065000, China

**Keywords:** transcriptomics, metabolome profiling, flavonoid metabolism, *Acanthus ilicifolius*, medicinal value

## Abstract

*Acanthus ilicifolius* is an important medicinal plant in mangrove forests, which is rich in secondary metabolites with various biological activities. In this study, we used transcriptomic analysis to obtain differentially expressed genes in the flavonoid metabolic pathway and metabolomic methods to detect changes in the types and content in the flavonoid metabolic synthesis pathway. The results showed that DEGs were identified in the mature roots vs. leaves comparison (9001 up-regulated and 8910 down-regulated), mature roots vs. stems comparison (5861 up-regulated and 7374 down-regulated), and mature stems vs. leaves comparison (10,837 up-regulated and 11,903 down-regulated). Furthermore, two *AiCHS* genes and four *AiCHI* genes were up-regulated in the mature roots vs. stems of mature *A. ilicifolius*, and were down-regulated in mature stems vs. leaves, which were highly expressed in the *A. ilicifolius* stems. A total of 215 differential metabolites were found in the roots vs. leaves of mature *A. ilicifolius*, 173 differential metabolites in the roots vs. stems, and 228 differential metabolites in the stems vs. leaves. The metabolomic results showed that some flavonoids in *A. ilicifolius* stems were higher than in the roots. A total of 18 flavonoid differential metabolites were detected in the roots, stems, and leaves of mature *A. ilicifolius*. In mature leaves, quercetin-3-O-glucoside-7-O-rhamnoside, gossypitrin, isoquercitrin, quercetin 3,7-bis-O-β-D-glucoside, and isorhamnetin 3-O-β-(2″-O-acetyl-β-D-glucuronide) were found in a high content, while in mature roots, di-O-methylquercetin and isorhamnetin were the major compounds. The combined analysis of the metabolome and transcriptome revealed that DEGs and differential metabolites were related to flavonoid biosynthesis. This study provides a theoretical basis for analyzing the molecular mechanism of flavonoid synthesis in *A. ilicifolius* and provides a reference for further research and exploitation of its medicinal value.

## 1. Introduction

*A. ilicifolius* Linn, a member of the family Acanthaceae, is a shrub or small tree that can reach up to two meters in height, with sturdy stalks. It grows in mangroves and tidal areas in tropical and subtropical regions [1,2]. Its unique growth environment results in a diverse array of structurally specific secondary metabolites, which exhibit a variety of biological activities and have potential medicinal value. The leaves, roots, and whole plant of *A. ilicifolius* are used in traditional medicine in India and China. It is considered an important medicinal plant in mangroves.

The properties of *A. ilicifolius* in traditional Chinese medicine are described as cold and mild in nature [3]. It is considered effective for clearing heat and detoxifying, eliminating swelling and dispersing knots, and relieving cough and asthma [4]. In China, India, and Southeast Asian countries such as Thailand, *A. ilicifolius* is used to treat conditions including neuralgia, rheumatism, snakebite, and paralysis [5]. In Thai folk medicine, the Acanthus genus is commonly used as a laxative, anti-inflammatory treatment for rheumatoid arthritis, antipyretic, anti-abscess, and anti-ulcer agent. Its leaves are used to treat rheumatism, snakebite, paralysis, and asthma. It is often used in combination with pepper as a tonic [6]. Pharmacological studies have shown that *A. ilicifolius* has hepatoprotective, antioxidant, antitumor, antibacterial, and antiviral effects [5,7,8]. Its chemical composition is mainly benzoxazin and benzoxazolinone alkaloids [9]. Further studies have revealed that it contains various types of compounds, such as flavonoids, triterpenoids, lignans, phenylethanoid glycosides, and sterols [5]. *A. ilicifolius* contains a large number of biologically active flavonoids, and the total flavonoid content can reach 3.82% [10]. In our study, the total flavonoid content of *A. ilicifolius* roots and stems reached 4.07 mass % (Figure S1).

Flavonoids are a diverse class of polyphenolic compounds [11] exhibiting a wide range of molecular structures and biological activities. They are derived from the metabolic pathway of phenylpropanoid and can be classified into seven major groups: flavonoids, flavonols, flavanones, anthocyanins, flavanols, isoflavones, and proanthocyanidins [12]. The basic backbone of flavonoids is a C6-C3-C6 diphenylpropane structure consisting of two benzene rings (A and B rings) interconnected with three carbon atoms in the center [11]. Flavonoids have been shown to possess various pharmacological effects and biological activities, such as anti-bacterial, anti-inflammatory, anti-oxidant, anti-aging, detoxifying, anti-cancer, and cardio- and cerebrovascular protective effects [13,14,15,16].

The biosynthesis of flavonoids is a crucial area of research in the secondary metabolism of plants [17]. The flavonoid metabolic pathway is a complex and multifactor-regulated network [18,19]. The phenylalanine metabolic pathway represents an important metabolic pathway [18,20], and a large number of secondary metabolites are produced from phenylalanine or tyrosine, including flavonoids and isoflavonoids, monolignans, various phenolic acids and stilbenes [20]. The biosynthesis of plant flavonoids is predominantly governed by the concerted action of structural and regulatory genes. Structural genes encode key enzymes that catalyze the flavonoid biosynthesis pathway, such as phenylalanine deaminase, cinnamic acid-4-hydroxylase, and 4-coumarate coenzyme-A-ligase. The activity of these structural enzymes and their gene expression levels directly impact the flavonoid content in the biosynthetic pathway. However, flavonoids are evolutionarily conserved in plants, and studying their metabolic pathways and regulatory mechanisms in different plant species is of significant biological importance [19].

The current study of the biosynthesis and accumulation of secondary metabolites has made substantial progress. Still, little is known about the effects of developmental and environmental factors on the synthesis and accumulation of secondary metabolites in medicinal plants [21]. In this study, we utilized transcriptomics and metabolomics approaches to analyze differential gene and metabolite expression in the roots, stems, and leaves of *A. ilicifolius* at various developmental stages. We aimed to identify key genes closely associated with flavonoid biosynthesis, elucidate the molecular mechanisms underlying flavonoid synthesis in *A. ilicifolius*, and analyze the metabolite types and accumulation patterns. The ultimate goal was to provide a reference for further research and exploitation of the medicinal value of *A. ilicifolius*’ s flavonoid products.

## 2. Materials and Methods

### 2.1. Plant Material

*A. ilicifolius* plants were collected from the shore of the Dangjiang River in Beihai, Guangxi (21.595° N, 109.081° E), and identified by the experts from the Beihai Mangrove Breeding Base. Their young roots, stems, and leaves (mRY, mSY, and mLY) and old/mature roots, stems, and leaves (mRO, mSO, and mLO) were sampled in April 2020, treated with liquid nitrogen, and then stored at −80 °C for total RNA extraction. To ensure the samples’ consistency, *A. ilicifolius* plants were collected from the same locations, dried, and used for chemical composition analysis. Three biological replicates were set up for each sample. In this paper, mRY, mSY, and mLY represent the young roots, young stems, and young leaves, respectively, and mOR, mOS, and mOL, represent the mature roots, mature stems, and mature leaves, respectively.

### 2.2. Transcription Experimental Methods

The total RNA was isolated from the young and mature tissues of roots, stems, and leaves with three biological replicates using FastPure Cell/Tissue Total RNA Isolation Kit (Vazyme, Nanjing, China). The RNA concentration was detected by Nanodrop. Agilent 2100 Bioanalyzer (Agilent Technologies, Santa Clara, CA, USA) was employed for detecting RIN (RNA Integrity Number) and 28S/18S values. Then, RNA integrity and quality were measured using 1% agarose electrophoresis. 

Eukaryotic mRNA was enriched with magnetic beads with Oligo (dT), and the mRNA was randomly interrupted by adding Fragmentation Buffer; the first cDNA strand was synthesized with six-base random hexamers using mRNA as a template, and the second cDNA strand was synthesized by adding buffer, dNTPs, RNase H, and DNA polymerase I. The purified double-stranded cDNA was then end-repaired, A-tailed, and connected to sequencing junctions, followed by fragment size selection with AMPure XP beads; finally, the cDNA library was enriched by PCR. Sequencing was performed with the Illumina novaseq 6000 platform (Illumina, San Diego, CA, USA).

Before data analysis, the quality of raw reads was checked using FastQC (http://www.bioinformatics.babraham.ac.uk/projects/fastqc/, accessed on 10 November 2022). The impure reads were filtered, whereas the high-quality ones were acquired using Trinity by adopting default parameters, which were later utilized for constructing the distinct consensus sequences [22].

The filtered reads were first localized to the genome. The localized reads on the genome matched transcripts and the specified *A. ilicifolius* genome (unpublished data, Genome submission: SUB12869169) were used as a reference for sequence alignment and subsequent analysis in this project.

FPKM (fragments per kilobase of transcript per million fragments mapped) values [23] were estimated to measure gene expression by Cufflinks [24]. DESeq2 [25] was used to identify differentially expressed genes (DEGs). During differentially expressed gene detection, Fold Change ≥ 1.5 and *p* value < 0. 01 were used as screening criteria during the expressed gene detection [26].

Subsequently, the obtained genes were mapped against NR (Non-redundant protein sequence database in GenBank) [27], Swiss-Prot (Protein Sequence Database) [28], and COG (Cluster of Orthologous Groups of proteins) [29], KOG (Clusters of orthologous groups for eukaryotic complete genomes) [30] and KEGG (Kyoto Encyclopedia of Genes and Genomes) databases were used for sequence comparison [31].

### 2.3. Metabolome Analysis Methods

The samples were freeze-dried in a freeze-dryer (Scientz-100F, Ningbo Xinzhi Freeze Drying Equipment Co., Ningbo, China), and each sample was accurately weighed to 0.1 g after grinding and was dissolved in 0.6 mL of 70% methanol extract. The dissolved samples were refrigerated at 4 °C overnight, during which the samples were vortexed six times to improve the extraction rate. After centrifugation of the liquid (10,000× *g*, 10 min), the supernatant was aspirated, and the samples were filtered through a microporous membrane (0.22 μm pore size) and stored in a feed bottle for UPLC-MS/MS analysis [32,33]. Three biological replicate samples and four mixed samples for quality control were set up for widely targeted metabolome analysis.

### 2.4. UPLC Conditions

The data acquisition instrumentation system consisted mainly of Ultra Performance Liquid Chromatography (UPLC) (Shim-pack UFLC SHIMADZU CBM30A, https://www.shimadzu.com.cn/, accessed on 13 November 2022) and tandem mass spectrometry (Tandem mass spectrometry, MS/MS) (Applied Biosystems 4500 QTRAP, https://www.shimadzu.com.cn/, accessed on 25 November 2022). Tandem mass spectrometry (MS/MS) (Applied Biosystems 4500 QTRAP, http://www.appliedbiosystems.com.cn/, accessed on 14 December 2022).

Liquid chromatography was performed using a Waters ACQUITY UPLC HSS T3 C18 (Waters, Milford, MA, USA) 1.8 µm, 2.1 mm × 100 mm column. The mobile phase consisted of ultra-pure water for phase A (with 0.04% acetic acid added) and acetonitrile for phase B (with 0.04% acetic acid added). The elution gradient involved a B-phase ratio of 5% at 0.00 min, which increased linearly to 95% within 10.00 min and was maintained at 95% for 1 min. From 11.00 to 11.10 min, the B-phase ratio decreased to 5% and equilibrated with 5% until 14 min. The flow rate was 0.35 mL/min, and the column temperature was set to 40 °C. The injection volume was 4 μL.

The mass spectrometry conditions used in this study involved electrospray ionization (ESI) at 550 °C, mass spectrometry at 5500 V, curtain gas (CUR) at 30 psi, collision-activated dissociation (CAD), and a mass spectrometer at 5000 V. A high parameter was set for each analysis. In the triple quadrupole (QQQ) mass spectrometer, each ion pair was scanned and detected using optimized declustering potential (DP) and collision energy (CE) values, as previously described by Chen et al. [34].

Specific fragment ions were compared to the reference for identifying secondary metabolites and additional amino acids [35]. Metabolites that shared close fragment ions were deemed to be identical compounds. Statistical analysis of secondary metabolite data was performed using Analyst 1.6.1 software (AB SCIEX, Framingham, MA, USA). Variable importance in projection (VIP) values were determined through partial least squares discriminant analysis. The differentially changed metabolites (DCMs) were selected based on the thresholds of VIP ≥ 1 and absolute Log2FC (fold change) ≥ 1. Identified metabolites were annotated using the KEGG Compound database (http://www.kegg.jp/kegg/compound/, accessed on 15 January 2023), and annotated metabolites were mapped to the KEGG Pathway database (http://www.kegg.jp/kegg/pathway.html, accessed on 21 January 2023). Pathways with significantly regulated metabolites were mapped into MSEA (metabolite sets enrichment analysis), and their significance was determined by the hypergeometric test’s *p*-values.

### 2.5. qRT-PCR

Real-time quantitative reverse transcription PCR (qRT-PCR) was performed using the TUREscript first Stand cDNA SYNTHESIS Kit instructions (Edelweiss). The nine genes were validated by qRT-PCR using a thermal cycler (Analytikjena-qTOWER2.2, Jena, Germany). The 20 μL reverse transcription reaction system contained RNA (2 μL), 5 × RT reaction mixture (4 μL), Rondam primer/oligodT (50 pM, 0.8 μL), TUREscript H- RTase/RI mixture (200 U/Μl, 0.8 μL), and RNase-free ddH_2_O (12.4 μL). Reverse transcription reaction conditions: 42 °C for 40 min, 65 °C for 10 min, after the reaction, cDNA was obtained and stored at −80 °C. The 10 μL qPCR reaction system contained 2 × SYBR Green Supermix (1 × 0.5 μL), forward primer (10 μM, 0.5 μL), and reverse primer (10 μM, 0.5 μL), cDNA (N/A, 1 μL), and ddH_2_O (3 μL). The thermal profile was comprised of two segments: 30 s at 95 °C; 5 s denaturation at 95 °C, and 30 s annealing at 60 °C for a total of 40 cycles. Every assay was repeated thrice. Primer Express 2.0 Software (PE Applied Biosystems, Foster City, CA, USA) was applied in the primer design with default parameters. Supplementary Table S1 displayed the sequences of all the primers. Excel software (Microsoft Office, Redmond, WA, USA) and 2^−ΔΔCt^ [36] were used for data analysis, with the Actin gene as the reference.

## 3. Results

### 3.1. Transcriptomic Analysis and Differentially Expressed Genes

To explore genes related to flavonoid metabolism in *A. ilicifolius*, transcriptomic analysis was conducted on young and mature roots, stems, and leaves. Before transcriptome sequencing data were ready for subsequent analysis, quality control of raw data was required to obtain high quality data, also called clean reads. The overall clean read counts in each sample ranged from 20,006,083 to 29,156,661, producing a total of 123.5 Gb of clean data. The sequence reads were aligned to the reference genome of *A. ilicifolius*, with more than 84.65% of reads mapped successfully. The high-quality transcriptomic results were supported by a GC concentration of 47% and a Q30 score of over 93.39% (Table S2).

**Figure 1:**
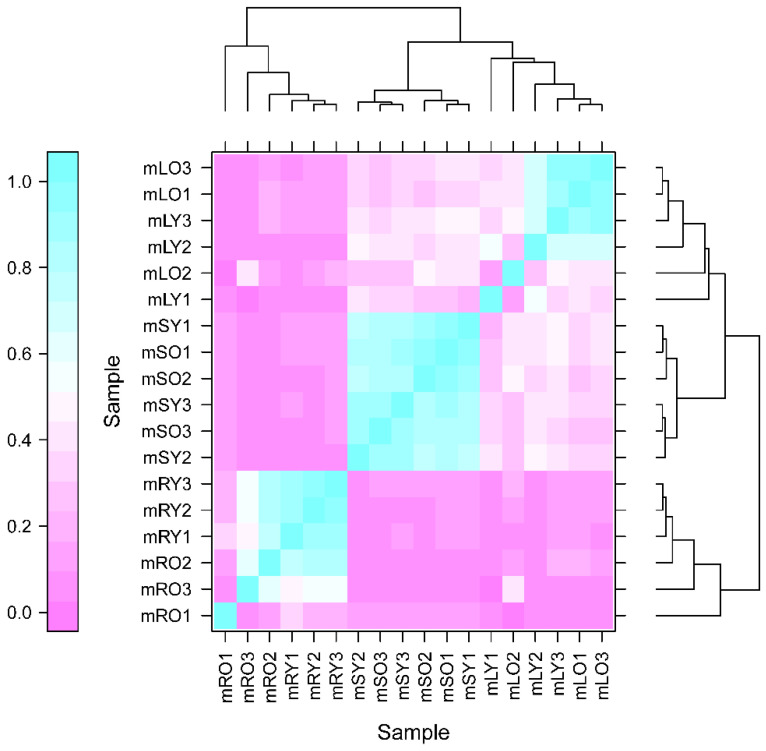
Heatmap of the expression correlation between two samples. Young roots, young stems, and young leaves are represented by mRY, mSY, and mLY, respectively, and mature roots, mature stems, and mature leaves are represented by mOR, mOS, and mOL, respectively. The numbers after the sample names indicate the plant numbers from different biological replicates.

The high sensitivity of transcriptomic data enabled the detection of the gene expression levels. In this study, protein-coding genes with expression levels represented as FPKM values were sequenced and found to span six orders of magnitude, ranging from 0.01 to 10,000 (Figure S2). Pearson’s Correlation Coefficient R (PCC) was used to assess biological repeat correlation and to screen for reliable differentially expressed genes. The r^2^ in this study was close to 1, indicating a strong correlation of duplicate samples, which facilitated the follow-up analysis (Figure 1).

**Figure 2:**
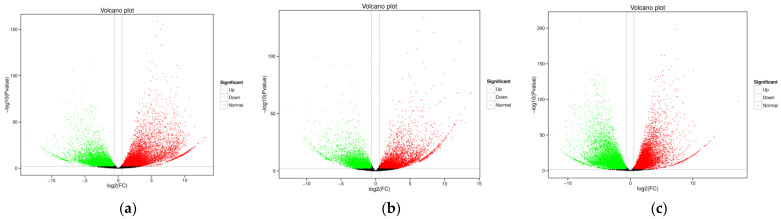
Volcano plots of the differential gene expression: (**a**) mRO vs. mLO; (**b**) mRO vs. mSO; (**c**) mSO vs. mLO. mOR, mOS, and mOL represent mature roots, mature stems, and mature leaves, respectively.

This study focused on flavonoids, a class of secondary metabolites. Flavonoid accumulation was higher in the roots, stems, and leaves of mature *A. ilicifolius*, prompting an analysis of the DEGs in these organs. In mature *A. ilicifolius*, a total of 9001 DEGs were up-regulated and 8910 DEGs were down-regulated in the mature roots vs. leaves comparison (Figure 2a). Similarly, 5861 DEGs were up-regulated and 7374 DEGs were down-regulated in the mature roots vs. stems comparison (Figure 2b), and 10,837 DEGs were up-regulated and 11,903 DEGs were down-regulated in the mature stems vs. leaves comparison (Figure 2c).

**Figure 3:**
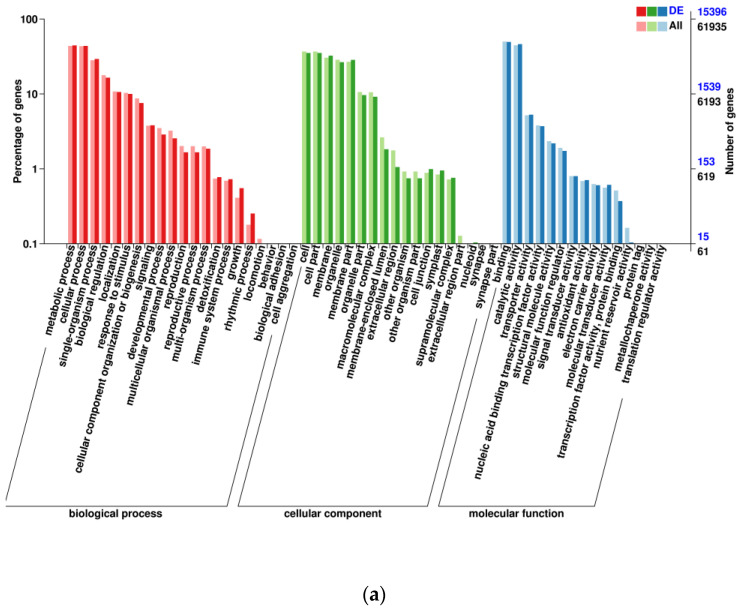

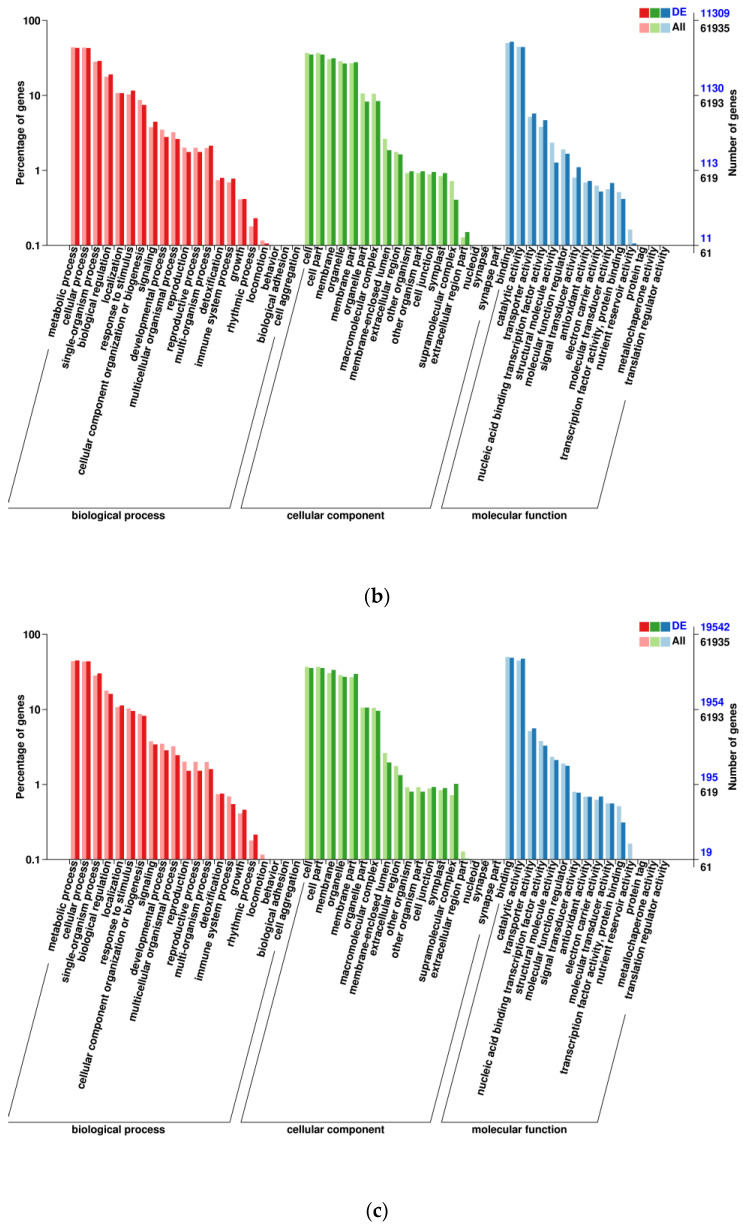
Statistical graph of GO annotation classification of differentially expressed genes. (**a**) mRO vs. mLO; (**b**) mRO vs. mSO; (**c**) mSO vs. mLO. mOR, mOS, and mOL represent mature roots, mature stems, and mature leaves, respectively.

**Figure 4:**
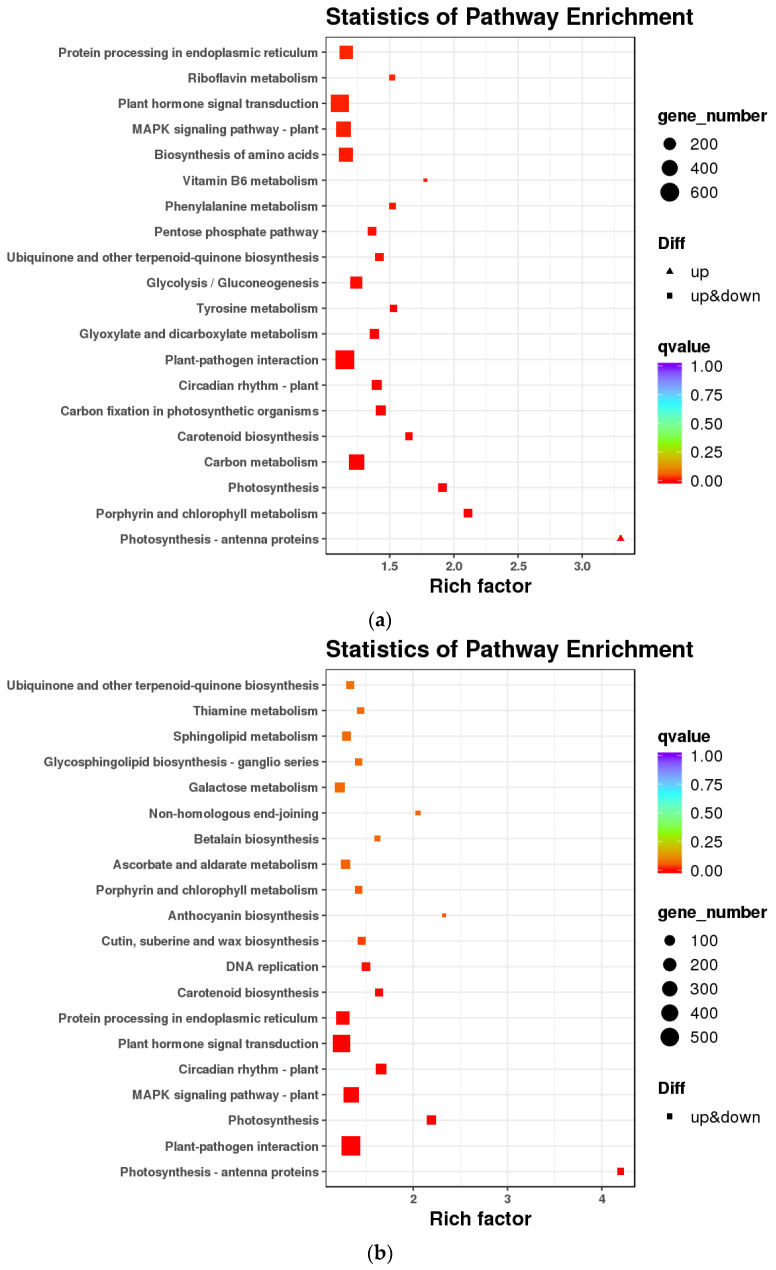

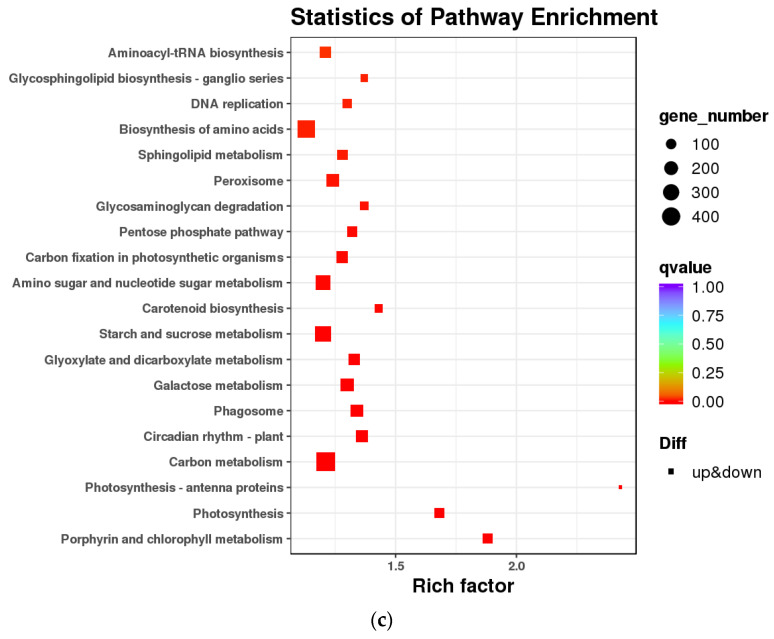
Enrichment scatters plot of differentially expressed genes in the KEGG pathway. (**a**) mRO vs. mLO; (**b**) mRO vs. mSO, (**c**) mSO vs. mLO. mOR, mOS, and mOL represent mature roots, mature stems, and mature leaves, respectively.

DEGs in mature roots vs. leaves, roots vs. stems, and stems vs. leaves of *A. ilicifolius* were mainly enriched in metabolic processes, single-organism process, cell, cell part membrane, organelle, binding, and catalytic activity (Figure 3). KEGG enrichment further indicated that the DEGs of mature roots vs. leaves and roots vs. stems of *A. ilicifolius* were mainly enriched in the plant–pathogen interaction and plant hormone signal transduction (Figure 4a,b). KEGG enrichment further indicated that DEGs of mature stems vs. leaves were mainly enriched in carbon metabolism and the biosynthesis of amino acids (Figure 4c).

### 3.2. Flavonoid Biosynthesis Differential Genes

**Figure 5:**
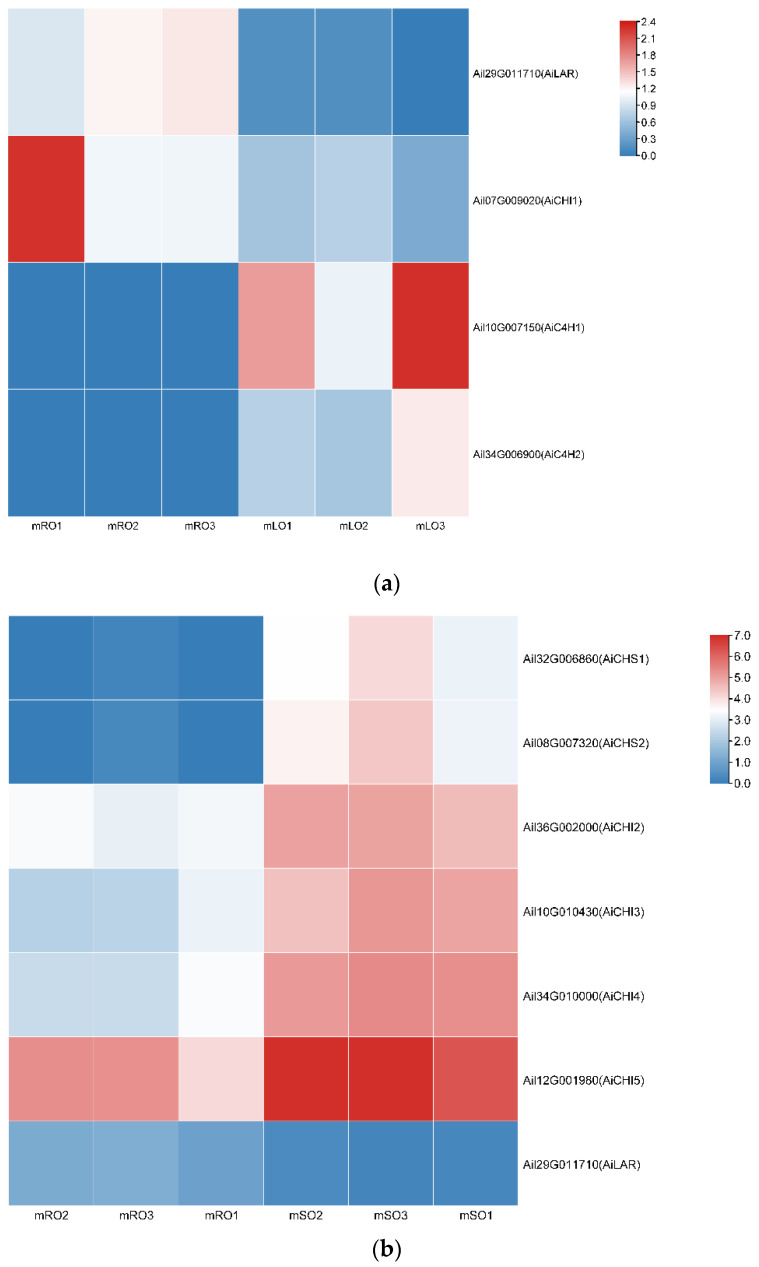

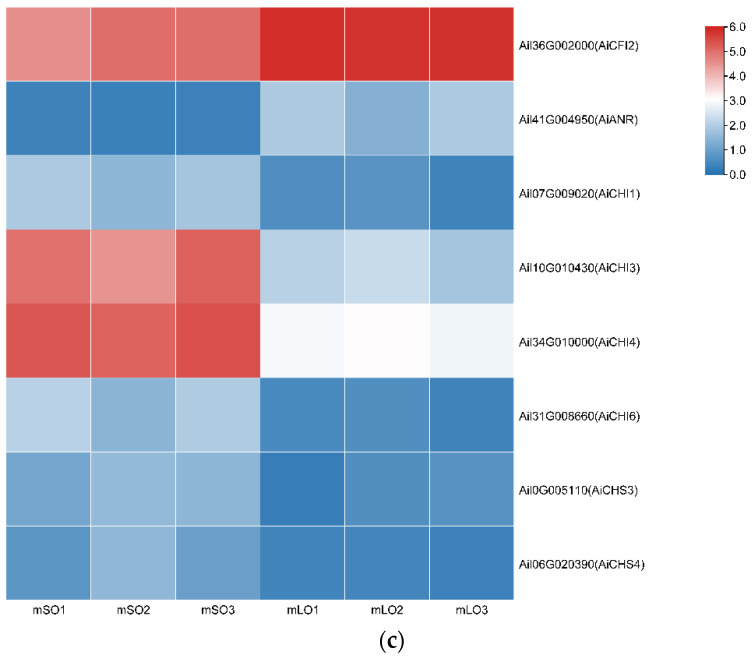
Differentially expressed genes related to flavonoid synthesis in *A. ilicifolius* parts. (**a**) mRO vs. mLO; (**b**) mRO vs. mSO, (**c**) mSO vs. mOR, mOS, and mOL represent mature roots, mature stems, and mature leaves, respectively. Each part has three biological replicates with the number following the sample name. The differential expression values of genes in the heatmap are treated with log2. Different *A. ilicifolius* genes and their related genes within the brackets are showed on the vertical axis.

In mature *A. ilicifolius*, four DEGs related to flavonoid biosynthesis were identified in the roots vs. leaves comparison, with two up-regulated and two down-regulated DEGs (Figure 5a). The up-regulated DEGs were two *AiC4H* genes (Ail10G007150 and Ail34G006900), which encode cinnamate 4-hydroxylase. The down-regulated DEGs were *AiLAR* (Ail29G011710), which encodes leucoanthocyantin reducase, and *AiCHI1* (Ail07G00902), which encodes chalcone isomerase. In the roots vs. stems comparison, seven flavonoid biosynthesis-related DEGs were identified, with six being up-regulated and one down-regulated (Figure 5b). The up-regulated DEGs were two *AiCHS* genes (Ail32G006860 and Ail08G007320), which encode chalcone synthase, and four *AiCHI* genes (Ail36G002000, Ail10G010430, Ail34G010000, and Ail12G001980), which encode chalcone isomerase. The down-regulated DEG was *AiLAR* (Ail29G011710). In the stems vs. leaves comparison, eight flavonoid biosynthesis-related DEGs were identified, with two being up-regulated and six down-regulated (Figure 5c). The up-regulated DEGs were *AiCFI2* (Ail36G002000), which encodes chalcone flavanone isomerase, and *AiANR* (Ail41G004950), which encodes anthocyanidin reductase. The down-regulated DEGs were four *AiCHI* genes (Ail07G009020, Ail10G010430, Ail34G010000, and Ail31G008660), which encode chalcone isomerase, and two *AiCHS* genes (Ail0G005110 and Ail06G020390), which also encode chalcone synthase.

**Figure 6:**
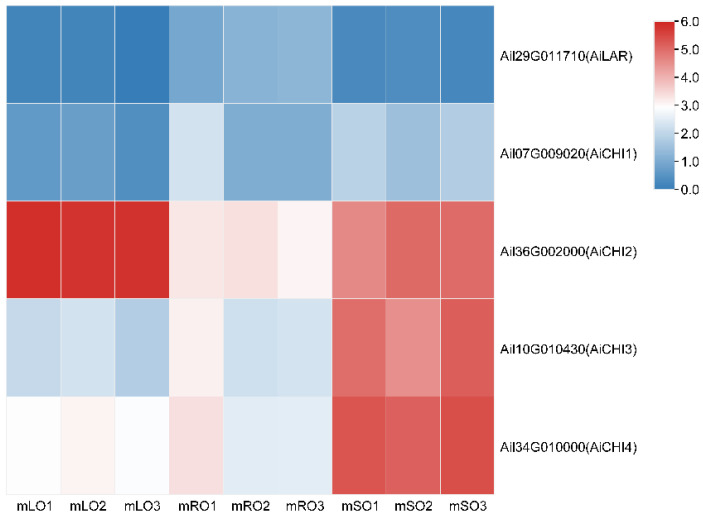
Expression heatmap of flavonoid synthesis-related differentially expressed genes in *A. ilicifolius* mature tissues. mOR, mOS, and mOL represent mature roots, mature stems, and mature leaves, respectively. The differential expression of genes in the heatmap are treated with log2.

The *AiLAR* gene encoding leucoanthocyanidin reductase was highly expressed in the mature roots of *A. ilicifolius* (Figure 6). *AiLAR* has been shown to reduce colorless anthocyanins and anthocyanins to flavanols, mainly catechin and epicatechin, and ultimately produce proanthocyanins through the processes of translocation, oxidation, and polymerization [37]. In contrast, *AiCHI* genes encoding chalcone isomerase were found to be highly expressed in the mature leaves and stems of *A. ilicifolius*. *CHI* catalyzes the specific cyclization of naringenin chalcone into naringenin, which is a common intermediate of several flavonoid subclasses, including flavonoids, flavanols, flavonols, anthocyanins, proanthocyanidins, terephthalic acid, and isoflavones [38].

### 3.3. qRT-PCR

**Figure 7:**
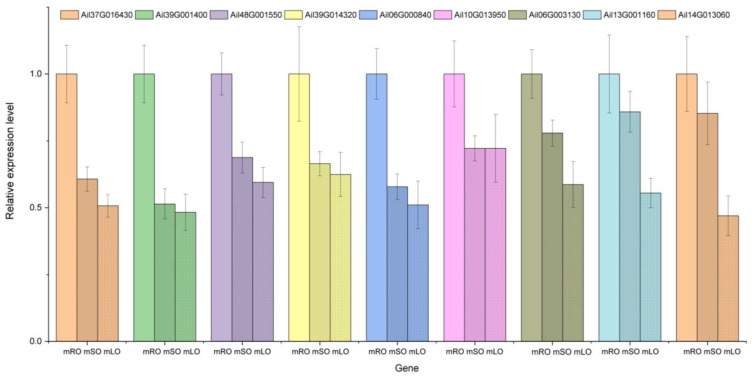
Expression levels of the candidate genes. The diagram distinguishes the nine genes with different colors. mOR, mOS, and mOL represent mature roots, mature stems, and mature leaves, respectively.

The nine DEGs were randomly selected, and transcriptional data showed that the gene expression was greater in the mature roots compared with the mature stems and lowest in mature leaves. These were further screened for qRT-PCR analysis, and the results were consistent with the transcriptome results (Figure 7).

### 3.4. Metabolome Analysis and Differential Metabolites

**Figure 8:**
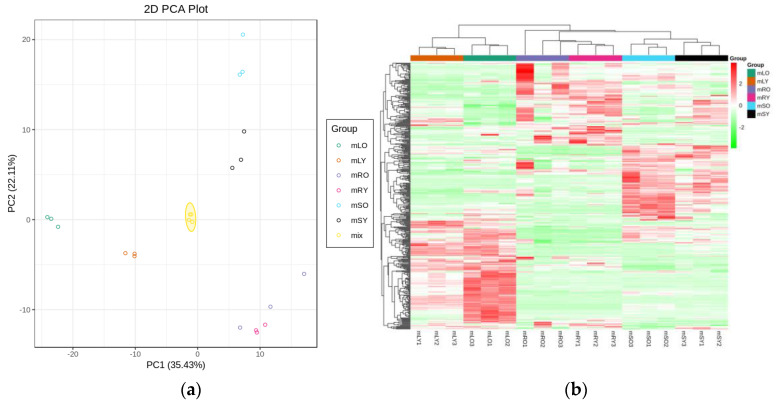
Analysis of *A. ilicifolius* metabolome samples. (**a**) Principal component analysis (PCA) of the young and mature root, stem, and leaf samples. The ordinate represents the clustering of samples, and the abscissa represents the clustering of metabolites. (**b**) The overall clustering map of samples is based on metabolite profiles. The horizontal coordinate indicates the sample name (hierarchical clustering result), and the vertical coordinate indicates all metabolites (hierarchical clustering result). mRY, mSY, and mLY represent young roots, young stems, and young leaves, respectively, and mOR, mOS, and mOL represent mature roots, mature stems, and mature leaves, respectively.

PCA analysis classified overall variation as PC1 and PC2, contributing 35.43% and 22.11%, respectively (Figure 8a). The correlations between samples with the same organ were high, indicating a good repeatability of samples, as well as stability and reliability of the experimental data. The results of the hierarchical clustering of metabolite profiles of different samples showed that the same parts from the same stage were basically clustered into one subclass, while the root, stem, and leaf samples were clustered into three major classes, respectively (Figure 8b). The results of the metabolome analysis revealed that the metabolites in the young and mature parts were different.

**Figure 9:**
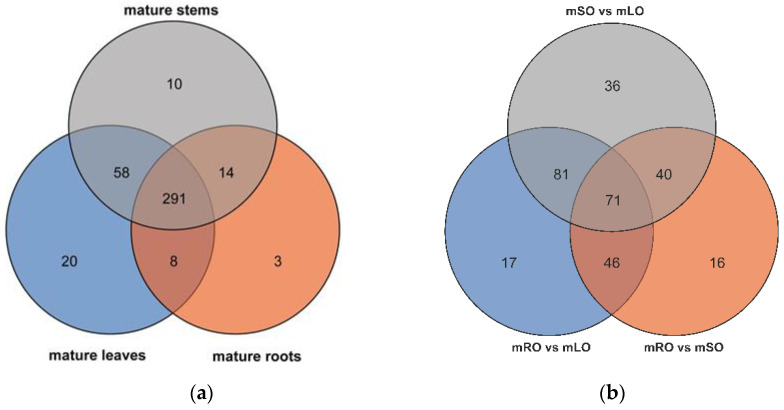
Venn diagram of the number of compounds. (**a**) Metabolites detected in the three mature parts. (**b**) Number of differential metabolites among the three mature parts. mOR, mOS, and mOL represent mature roots, mature stem, and mature leaves, respectively.

Figure 9a shows four mixed samples represent the quality, which were prepared from a mixture of sample extracts and used to analyze the reproducibility of the samples under the same processing method. In Figure 9b, the number after the sample name represents the different biological replicate number of samples.

**Figure 10:**
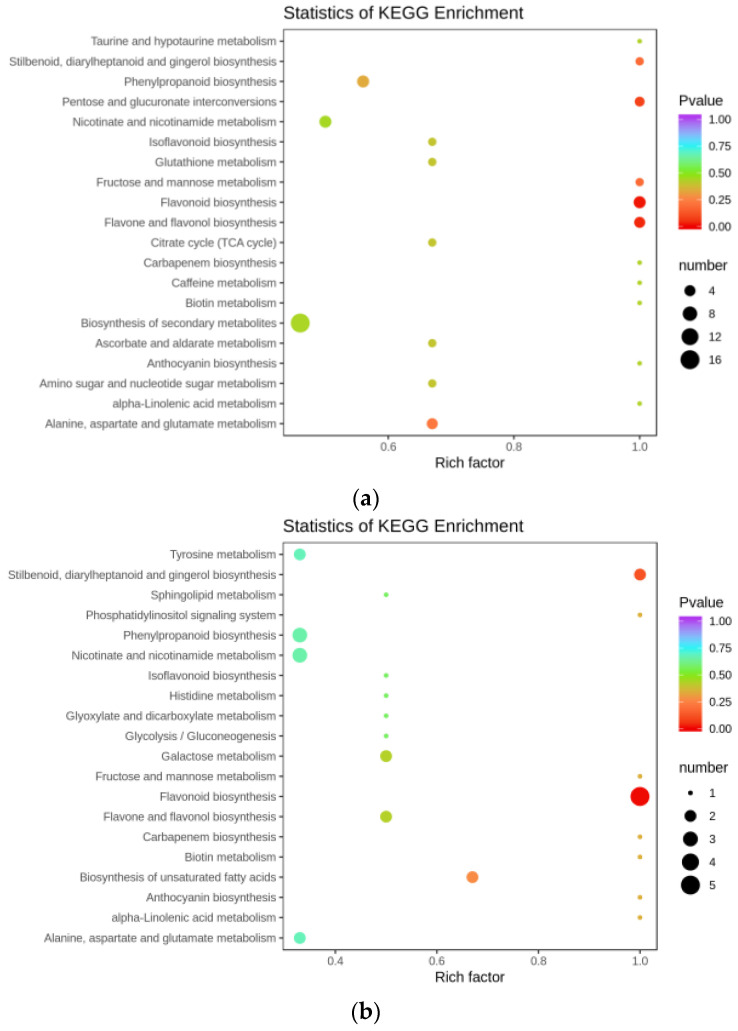

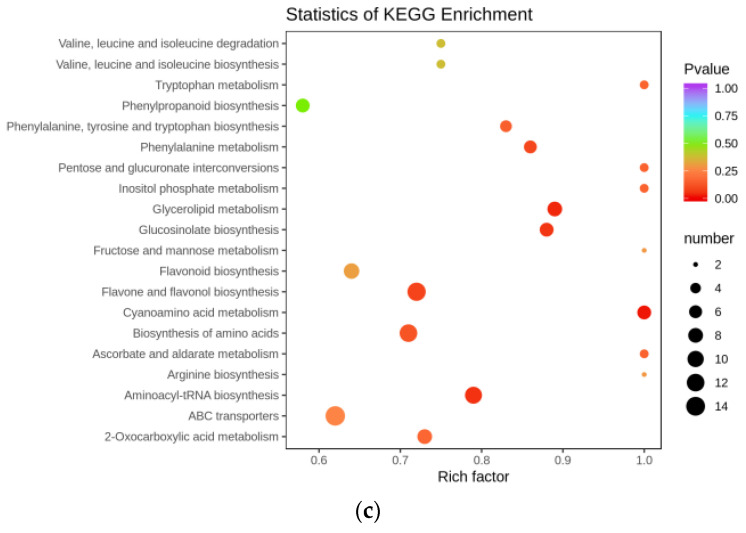
KEGG enrichment map of differential metabolites. (**a**) mRO vs. mLO; (**b**) mRO vs. mSO; (**c**) mSO vs. mLO. The horizontal coordinate indicates the rich factor of each pathway, the vertical coordinate is the pathway name, and the dot color is the p value; the redder it is, the more significant the enrichment. The size of the dots represents the number of differential metabolites enriched.

A total of 407 metabolites were detected based on the UPLC-MS/MS detection platform and the local metabolic database (Biomarker Technologies, Beijing, China) (Table S3). Our results indicate there were 377, 316, and 373 metabolites in total that were detected for mature leaf, root, and stem (Figure 9a) Of the 215 differential metabolites in the roots vs. leaves of mature *A. ilicifolius*, (Figure 9b) 68 were flavonoids (Table S4). There were 173 differential metabolites in the roots vs. stems of mature *A. ilicifolius*. Among them, there were 59 flavonoids (Table S5). There were 228 differential metabolites in the stems vs. leaves of mature *A. ilicifolius*. Among them were 59 flavonoids (Table S6). Furthermore, the KEGG pathway enrichment analysis showed that the significantly enriched pathways were a biosynthesis of the secondary metabolism, flavonoid biosynthesis, and flavone and flavonol biosynthesis in the roots and leaves (Figure 10a); flavonoid biosynthesis in the roots and stems (Figure 10b); and flavone and flavonol biosynthesis in the stems and leaves of mature *A. ilicifolius* (Figure 10c).

**Figure 11:**
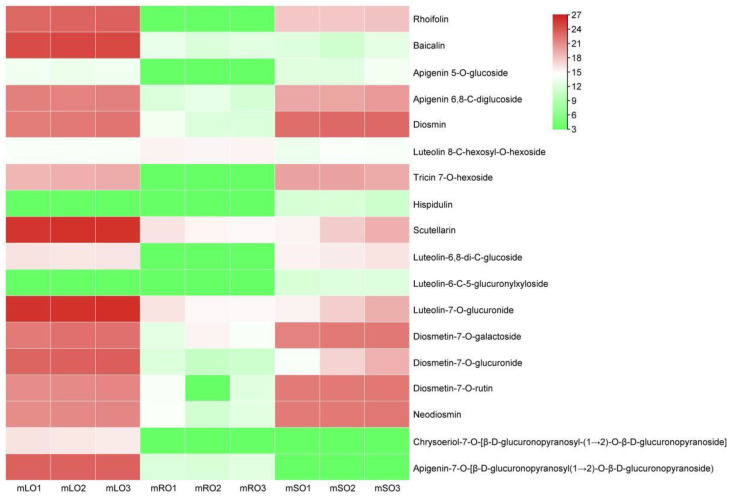
Flavonoid types and content heat map. Mature root, stem, and leaf samples are represented by mRO, mSO, and mLO, respectively. The flavonoid content in the heatmap is log2 processed.

A total of 18 differential flavonoid metabolites were detected in the mature leaves vs. roots of *A. ilicifolius* (Figure 11). In mature leaves, quercetin-3-O-glucoside-7-O-rhamnoside, gossypitrin, isoquercitrin (quercetin 3-O-β-D-glucoside), quercetin 3,7-bis-O-β-D-glucoside, and isorhamnetin 3-O-β-(2″-O-acetyl-β-D-glucuronide) were found in high amounts, while in mature roots, di-O-methylquercetin and isorhamnetin were the major compounds (Table 1).

In the mature stems and roots of *A. ilicifolius*, quercetin-3-O-glucoside-7-O-rhamnoside, isoquercitrin (quercetin 3-O-β-D-glucoside), and quercetin 3,7-bis-O-β-D-glucoside were present in high amounts in mature stems, while quercetin 3-O-rhanosylgalactoside and bioquercetin were found in high amounts in mature roots (Table 2).

In the mature stems and leaves of *A. ilicifolius*, quercetin-3-O-glucoside-7-O-rhamnoside, isoquercitrin (quercetin 3-O-β-D-glucoside), and isorhamnetin 3-O-β-(2″-O-acetyl-β-D-glucuronide) were present in high amounts in mature leaves, while quercitrin, di-O-methylquercetin, quercetin 3,7-bis-O-β-D-glucoside, and isorhamnetin were the major compounds in mature stems (Table 3).

### 3.5. Combined Analysis of Transcriptome and Metabolome Analysis

**Figure 12:**
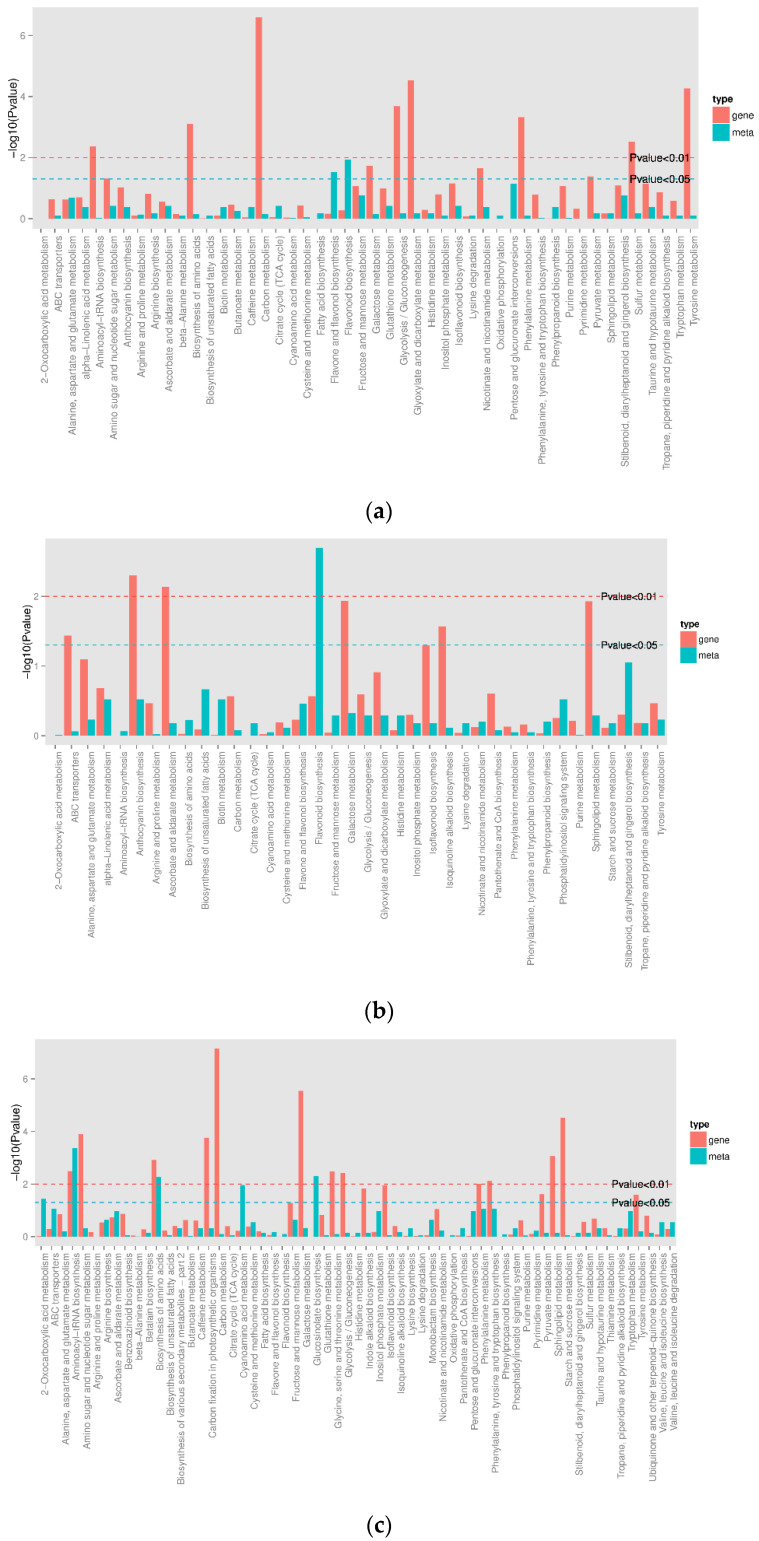
Column diagrams of differential metabolite and differential gene co-enrichment. (**a**) mRO vs. mLO; (**b**) mRO vs. mSO; (**c**) mSO vs. mLO.

The combined analysis of the metabolome and transcriptome revealed that DEGs and differential metabolites occurred in the roots and leaves of mature *A. ilicifolius*, and were found to be enriched in 48 metabolic pathways (Figure 12a) in the roots and stems of mature *A. ilicifolius*. In the stems and leaves of mature *A. ilicifolius,* 38 metabolic pathways (Figure 12b) and 61 metabolic pathways were enriched (Figure 12c). The differential metabolites and DEGs were associated with the flavonoid, flavone, and flavonol biosynthesis.

## 4. Discussion

### 4.1. Transcriptomic Analysis of Key Genes in the Flavonoid Synthesis Pathway in A. ilicifolius

Higher plants share a common core flavonoid pathway, while distinct species frequently create specialized branches to adapt to varying environmental situations [39]. Flavonoid anabolism begins with the metabolic pathway of phenylpropanoids. Phenylalanine deaminase catalyzes the conversion of phenylalanine to cinnamic acid, which is then hydroxylated to coumaric acid by cinnamic acid-4-hydroxylase [11]. Coumaric acid is converted to coumaroyl-CoA by 4-coumarate coenzyme A ligase. Chalcone synthase then catalyzes the condensation of coumaroyl-CoA and malonyl-CoA to produce chalcone, a precursor of various flavonoids. Subsequently, chalcone enters various branching pathways to produce different flavonoid classes via a series of enzymatic reactions [40]. In the present study, in *A. ilicifolius* mature roots vs. leaves, two AiC4H up-regulated genes, which played an important role in flavonoid synthesis, belong to the upstream genes.

The flavonoid synthesis pathway is broadly divided into two phases [41,42]. The pre-synthesis stage includes chalcone synthase (CHS), chalcone isomerase (CHI), and flavanone 3-hydroxylase (F3H), which are common genes involved in all downstream flavonoid biosynthesis pathways [43]. In this study, we found that two *AiCHS* genes and four *AiCHI* genes were up-regulated in mature roots vs. stems of mature *A. ilicifolius*, and down-regulated in stems vs. leaves, and these genes were highly expressed in *A. ilicifolius* stems. While CHS plays an important role in the first stage of flavonoid biosynthesis, where it catalyzes the stepwise condensation of 4-coumaryl-CoA and malonyl-CoA into naringenin chalcone [43]. 

Plant flavonoids perform various biological functions, such as protecting against UV radiation, protecting plants from pathogens and herbivores, regulating auxin transport, and signaling between microorganisms and plants, and are important pigments for flowers, fruits, seeds, and leaves [43,44,45]. The metabolomic results of this study showed that some flavonoids in *A. ilicifolius* stems were higher than those in roots and leaves, indicating that they played an important role in the synthesis of *A. ilicifolius* flavonoids.

Naringenin is a widely distributed flavonoid intermediate that serves as a precursor for various flavonoid subclasses, including anthocyanins, proanthocyanidins, terephthalic acid, and isoflavones [38]. Naringin flavanones are hydroxylated by flavanone 3-hydroxylase (F3H) to form dihydrosanninol [46], which is further hydroxylated by flavanone 3′-hydroxylase (F3′H) and flavanone 3′5′-hydroxylase (F3′5′H), to produce dihydroquercetin and dihydromyricetin. Dihydromyricetin, dihydroquercetin, and dihydromyricetin are dihydroflavonol compounds, representing new branch points in the flavonoid synthesis pathway. Dihydroflavonols are able to catalyze the formation of flavonols under the action of flavonol synthase (FLS) [47]. Alternatively, dihydroflavonols can be reduced to colorless anthocyanins by dihydroflavonol reductase (DFR) and NADPH [48]. Further oxidation by anthocyanin synthase (ANS) and modifications by various glycosylation modifying enzymes results in the formation of different types of anthocyanins [49]. Transcriptomic data from this study revealed that the *AiLAR* gene encoded leucoanthocyantin reductase was down-regulated in mature roots vs. leaves, and in mature roots vs. stems of mature *A. ilicifolius*, and the *AiANR* gene was up-regulated in mature roots vs. stems, indicating its potential role in flavonoid synthesis in *A. ilicifolius*.

Colorless anthocyanin reductase (LAR) and anthocyanin reductase (ANR) are capable of reducing colorless anthocyanins and anthocyanins to flavanolic substances, such as catechins and epicatechins, which can then be translocated, oxidized, and polymerized to form proanthocyanidins [37].

### 4.2. Metabolomic Analysis of Flavonoid Content in A. ilicifolius

*A. ilicifolius* contains many biologically active flavonoids, and the total flavonoid extraction can reach 3.82% [10]. Studies have shown that apigenin [5], apigenin-7-O-β-D-glucuronide [50], apigenin-7-O-β-D-glucuronide methyl ester, quercetin, quercetin-3-O-β-D-glucopyranoside [51,52], mucoxanthin, acanthin, lignan-7-O-β-D-glucuronide, 7-O-α-L-glucopyranoside [51,52], mucoxanthin-rhamnopyranoside-(1→6)-O-β-D-glucopyranoside [50,53], and other flavonoids.

In our study, 68 differential flavonoids were detected in the roots vs. leaves of mature *A. ilicifolius*, 59 differential flavonoids in the roots vs. stems of mature *A. ilicifolius*, and 59 differential flavonoids in the stems vs. leaves of mature *A. ilicifolius*. Flavonoids vary greatly in content and type in different species [54]. The content of flavonoid substances also varies in different tissues or developmental stages [55]. The study results show that 18 differential flavonoid metabolites were detected in the roots, stems, and leaves of mature *A. ilicifolius*, and the active ingredients of many traditional Chinese medicines were flavonoid substances [56]. 

We also found that quercetin-3-O-glucoside-7-O-rhamnoside, gossypitrin, isoquercitrin (Quercetin 3-O-β-D glucoside), quercetin 3,7-bis-O-β-D-glucoside, and isorhamnetin 3-O-β-(2″-O-acetyl-β-D-glucuronide) were high in mature *A. ilicifolius* leaves, and di-O-methylquercetin and isorhamnetin were high in mature *A. ilicifolius* roots. The natural substance quercetin could inhibit the proliferation of human nasopharyngeal carcinoma cells (CNE1), causing them to undergo natural apoptosis [57]. The isorhamnetin in the extract had antioxidant, antiplatelet, and anticoagulant effects and could be used for the prevention and treatment of cardiovascular diseases [58]. In mature *A. ilicifolius* leaves, quercetin-3-O-glucoside-7-O-rhamnoside, isoquercitrin (Quercetin 3-O-β-D-glucoside), and isorhamnetin 3-O-β-(2″-O-acetyl-β-D-glucuronide) were high, and quercitrin, di-O-methylquercetin, quercetin 3,7-bis-O-β-D-glucoside, and isorhamnetin were high in the stems. Sorhamnetin-3-O-rutinoside can induce apoptosis in chronic granulomatous leukemia cells K562 by activating the activity of apoptotic factors such as caspase-8 and caspase-3 [59].

These results suggest that different parts of the plant may have unique flavonoid profiles, which could have implications for their potential medicinal or commercial applications. In addition, the metabolomic analysis revealed that some flavonoids were found in specific tissues/parts.

Currently, limited research has been conducted on the mechanism underlying flavonoid biosynthesis in *A. ilicifolius*. In our study, gene upregulation and the accumulation of metabolites (as shown in Figure 12) were not associated with several biological processes. These indicate that the biosynthesis of flavonoids is a complex process that may involve feedback loops or other regulatory mechanisms. Additionally, it is possible that many factors can influence gene expression and metabolic pathways. For example, some metabolites may be produced in response to environmental stimuli rather than as a result of the gene expression [60]. Therefore, it is important to use multiple approaches to study biological processes, including for analyzing both the gene expression and metabolite levels [61]. 

In the present study, differentially expressed genes were identified through transcriptome analysis, and their impact on various substances in the metabolic pathway was assessed. In addition, metabolite types and content changes within the synthetic pathway were detected using metabolomic methods. These findings are crucial for comprehensively analyzing the mechanism underlying flavonoid biosynthesis in the medicinal *A. ilicifolius*. The findings of this study provide valuable insights into the biosynthesis and diversity of flavonoids in *A. ilicifolius*, which could have implications for the development of new medicinal or commercial products.

## 5. Conclusions

Our study focused on *A. ilicifolius*, a significant medicinal plant in mangrove forests with valuable biological activities. We employed transcriptomic and metabolomic methods to explore changes in the flavonoid synthesis pathway, which revealed significant findings. The transcriptomic analysis identified differentially expressed genes (DEGs) with more DEGs down-regulated than up-regulated. Two *AiCHS* genes and four *AiCHI* genes were up-regulated in the mature roots vs. stems of *A. ilicifolius*, which were highly expressed in the stems. Metabolomic analysis also revealed that differential metabolites were related to flavonoid biosynthesis. Our study identified the key genes closely associated with flavonoid biosynthesis, elucidated the molecular mechanisms underlying flavonoid synthesis in *A. ilicifolius*, and analyzed the metabolite types and accumulation patterns, and will provide a reference for further research and for exploitation of the medicinal value of *A. ilicifolius*’s flavonoid products.

## Figures and Tables

**Table 1:** Differential metabolites associated with flavonoid synthesis within the roots and leaves of mature *A. ilicifolius*.

Index	Formula	Compounds	VIP	Log2FC	Type
GQ512006	C_27_H_30_O_16_	Quercetin-3-O-glucoside-7-O-rhamnoside	1.24	16.60	up
mws1329	C_21_H_20_O_12_	Gossypitrin	1.17	1.24	up
pmb3894	C_17_H_14_O_7_	Di-O-methylquercetin	1.15	−1.59	down
pmp000580	C_21_H_20_O_12_	Isoquercitrin(Quercetin 3-O-β-D-glucoside)	1.24	19.40	up
pmp000596	C_27_H_30_O_17_	Quercetin 3,7-bis-O-β-D-glucoside	1.15	3.12	up
mws0066	C_16_H_12_O_7_	Isorhamnetin	1.21	−1.87	down
pmn001645	C_24_H_22_O_14_	Isorhamnetin 3-O-β-(2″-O-acetyl-β-D-glucuronide)	1.23	10.50	up

**Table 2:** Differential metabolites associated with flavonoid synthesis within the roots and stems of mature *A. ilicifolius*.

Index	Formula	Compounds	VIP	Log2FC	Type
GQ512006	C_27_H_30_O_16_	Quercetin-3-O-glucoside-7-O-rhamnoside	1.32	12.80	up
Li512117	C_27_H_30_O_16_	Quercetin 3-O-rhanosylgalactoside	1.19	−1.01	down
pmn001583	C_27_H_30_O_16_	Bioquercetin	1.20	−1.20	down
pmp000580	C_21_H_20_O_12_	Isoquercitrin(Quercetin 3-O-β-D-glucoside)	1.32	14.20	up
pmp000596	C_27_H_30_O_17_	Quercetin 3,7-bis-O-β-D-glucoside	1.28	4.81	up

**Table 3:** Differential metabolites associated with flavonoid synthesis within stems and leaves of mature *A. ilicifolius*.

Index	Formula	Compounds	VIP	Log2FC	Type
GQ512006	C_27_H_30_O_16_	Quercetin-3-O-glucoside-7-O-rhamnoside	1.16	3.84	up
mws0045	C_21_H_20_O_11_	Quercitrin	1.07	−1.85	down
pmb3894	C_17_H_14_O_7_	Di-O-methylquercetin	1.11	−2.21	down
pmp000580	C_21_H_20_O_12_	Isoquercitrin(Quercetin 3-O-β-D-glucoside)	1.16	5.22	up
pmp000596	C_27_H_30_O_17_	Quercetin 3,7-bis-O-β-D-glucoside	1.08	−1.69	down
mws0066	C_16_H_12_O_7_	Isorhamnetin	1.14	−1.09	down
pmn001645	C_24_H_22_O_14_	Isorhamnetin 3-O-β-(2″-O-acetyl-β-D-glucuronide)	1.17	10.5	up

## Data Availability

The transcriptome raw read data that support the findings of this study are available in the NCBI BioProject database (transcriptome raw read data, SRA submission: SUB12510474; and *A. ilicifolius* genome sequence, genome submission: SUB12869169).

## Supplementary Materials

The following supporting information can be downloaded at: https://www.mdpi.com/article/10.3390/genes14030752/s1. Figure S1: Total flavonoid content of roots, stems, and leaves of *A. ilicifolius*. Figure S2: Comparison of FPKM density distribution of each sample. Table S1: Sequences of primers. Table S2: RNA sequencing data and quality control. Table S3: All metabolites detected in roots, stems, and leaves of *A. ilicifolius*. Table S4: Differential metabolites in the roots vs. leaves of mature *A. ilicifolius*. Table S5: Differential metabolites in the roots vs. stems of mature *A. ilicifolius*. Table S6: Differential metabolites in the stems vs. leaves of mature *A. ilicifolius*.
