# Supplementary material for: Transcriptome and Metabolome Profiling Provide Insights into Flavonoid Synthesis in Acanthus ilicifolius Linn
Source: Genes (Basel). 2023 Mar 20;14(3):752. doi: 10.3390/genes14030752 (PMC10048380; doi:10.3390/genes14030752)
Supplement: Supplementary file 1 [file genes-14-00752-s001.zip › Figure S1.pdf]

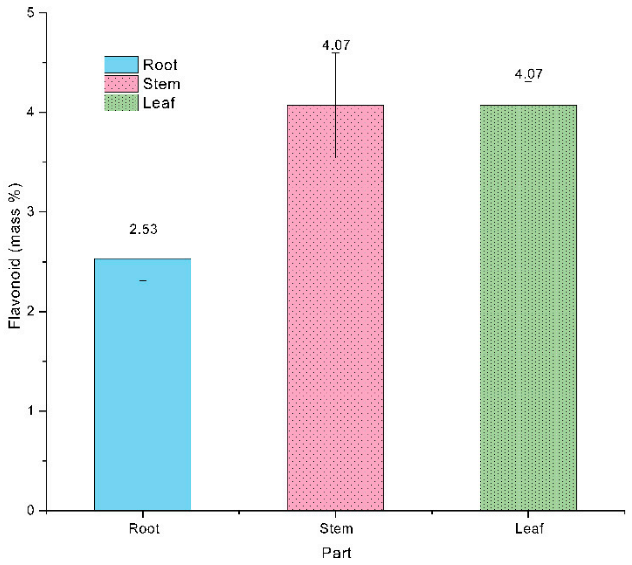

Figure S1. Total flavonoid content of roots, stems and leaves of *A. ilicifolius*. The determination of total flavonoid content was determined by the colorimetric method. The absorbance of flavonoids was measured at a wavelength of 506 nm, utilizing a visible spectrophotometer (PE Lambda-6, USA). The total flavonoid concentrations were calculated based on the linear equations derived from catechin (purity >97.50%, Macklin Inc, Shanghai) standard solutions of various concentrations. The equation obtained was y = 1.2051x-0.0066 (R^2 = 0.9988, SEE = 0.0119).
