# Supplementary material for: Transcriptome and Metabolome Profiling Provide Insights into Flavonoid Synthesis in Acanthus ilicifolius Linn
Source: Genes (Basel). 2023 Mar 20;14(3):752. doi: 10.3390/genes14030752 (PMC10048380; doi:10.3390/genes14030752)
Supplement: Supplementary file 1 [file genes-14-00752-s001.zip › Figure S2.pdf]

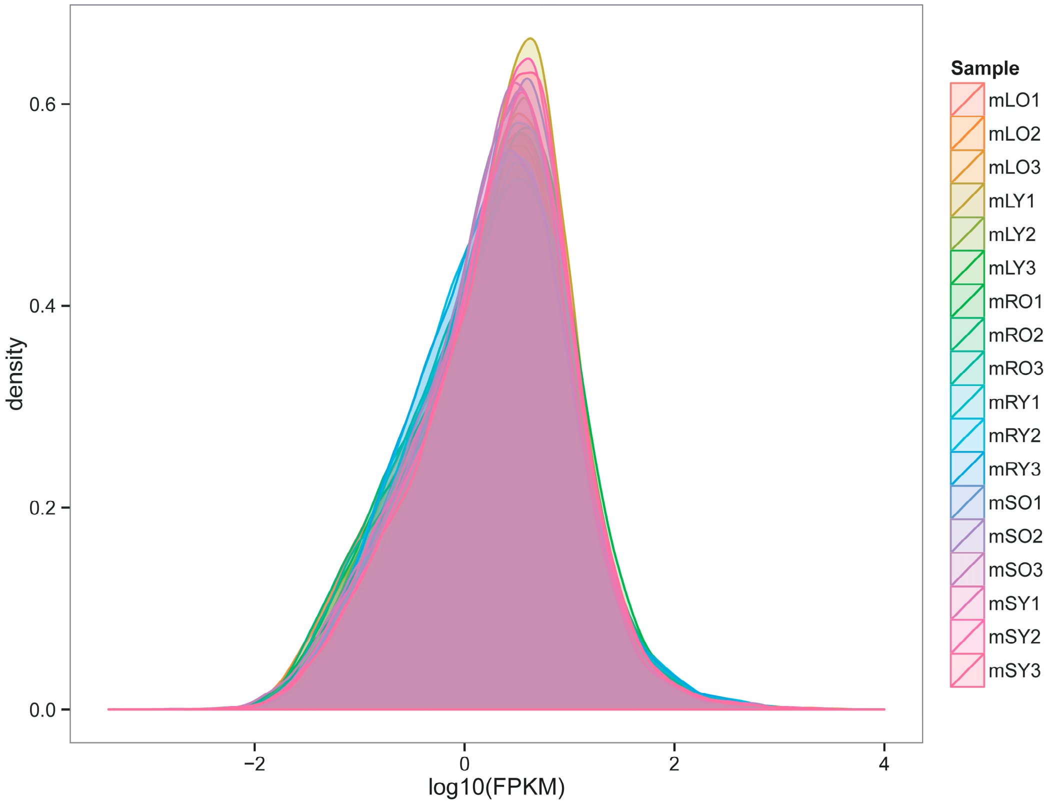


**Figure S2.** Comparison of FPKM density distribution of each sample. Young roots, young stems, and young leaves are represented by the mRY, mSY, and mLY, respectively, and mature roots, mature stems, and mature leaves are represented by the mOR, mOS, and mOL, respectively.
